# Squamoid Eccrine Ductal Carcinoma: An Unusual Diagnosis in a Pregnant Patient

**DOI:** 10.7759/cureus.72123

**Published:** 2024-10-22

**Authors:** Joshua A Bellisario, Alicia Lunardhi, Kimberly Huynh, Chika Iguh, Hindi Stohl

**Affiliations:** 1 Obstetrics and Gynecology, Harbor UCLA Medical Center, Torrance, USA; 2 Pathology, Harbor UCLA Medical Center, Torrance, USA

**Keywords:** pregnancy cancer, rare skin lesion, ­skin cancer, skin condition pregnancy, squamoid eccrine ductal carcinoma

## Abstract

Squamoid eccrine ductal carcinoma (SEDC) is an unusually rare neoplasm of the skin with a relatively high risk for local recurrence and a potential for metastasis. While typical presentations occur in older, male patients, this case report describes the diagnosis made in a pregnant patient in her third trimester. The clinical presentation, pathology, and treatment course of SEDC are outlined in this article.

## Introduction

Eccrine carcinomas, or sweat gland tumors, make up less than 0.01% of skin neoplasms, leading to limited clinical exposure from those who are just beginning their medical training to even the most senior physicians [1]. A subtype of these tumors, eccrine ductal carcinomas, contains several phenotypic variations. These include a variant containing fibromyxoid stroma, a basaloid variant, a spindle cell variant with myoepithelial differentiation, and one with squamous differentiation [1,2]. 

Squamoid eccrine ductal carcinoma (SEDC) is an unusually rare neoplasm of the skin with a relatively high risk for local recurrence and a potential for metastasis [3]. Presentations of this lesion can vary widely from simple plaques to ulcerated nodules, and to the untrained eye, the diagnosis can easily be overlooked as a different type of skin cancer or even a skin infection [4]. Complicating matters, SEDC is often misdiagnosed, as superficial shave biopsies may not collect the complete makeup of cells necessary to correctly identify the lesion [3,5]. While the chance of distant spread is low, aggressive metastasis have been reported, and given the high rate of misdiagnosis, this number could be much higher [6]. The median age of diagnosis is 80 years old, with the disease being more common in males [4]. As far as we are aware, this is the first known case report describing the development of SEDC in a pregnant woman. Given the overall rarity of this neoplasm, this is an important case report not only for dermatologists and pathologists who regularly care for dermatoses but also for helping the obstetrics care provider to keep differential diagnoses broad when evaluating dermatologic issues in pregnancy. 

## Case presentation

**Figure 1:**
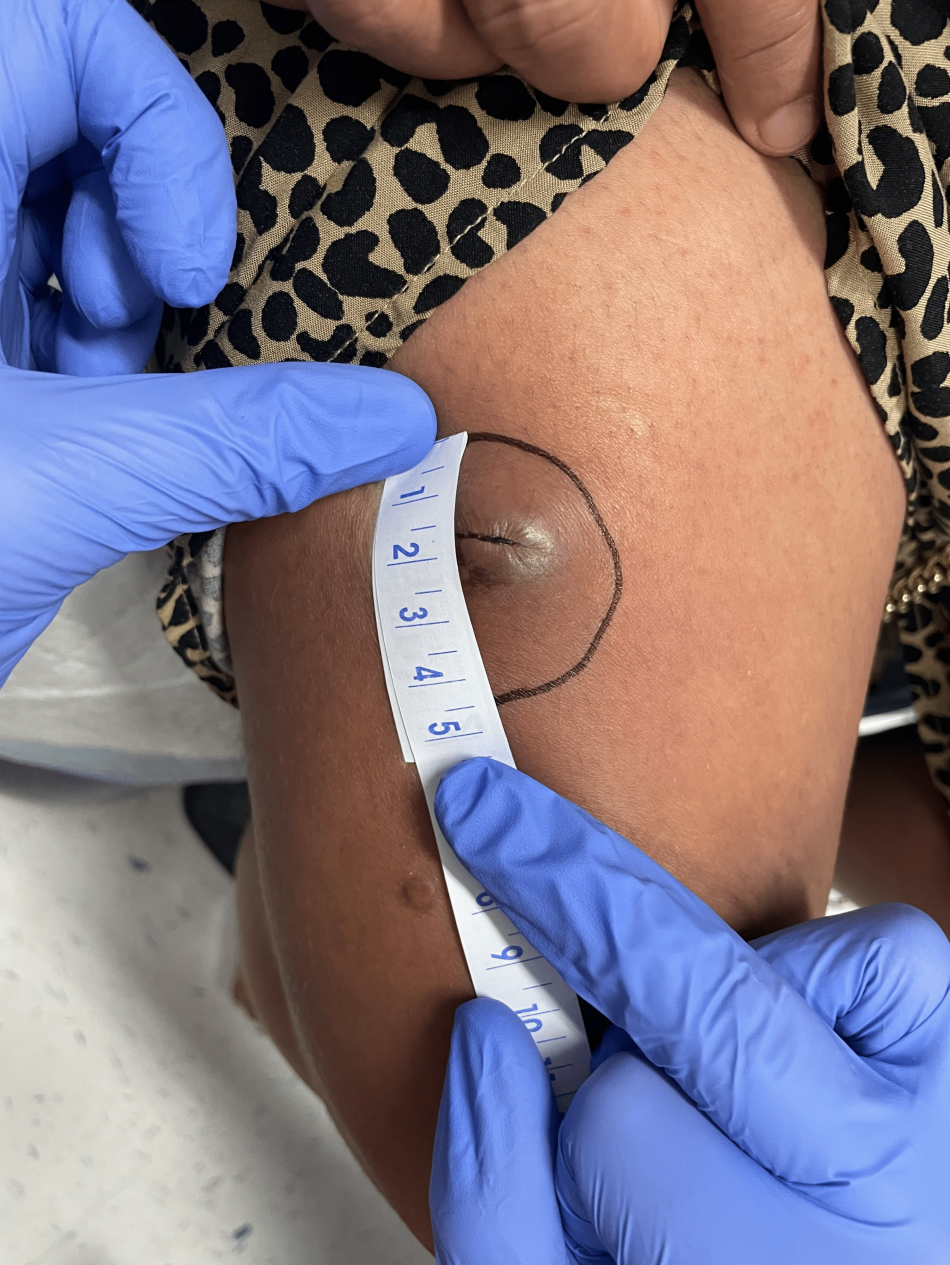
Photograph of lesion on the right thigh six days after initial attempt to drain and biopsy.

**Figure 2:**
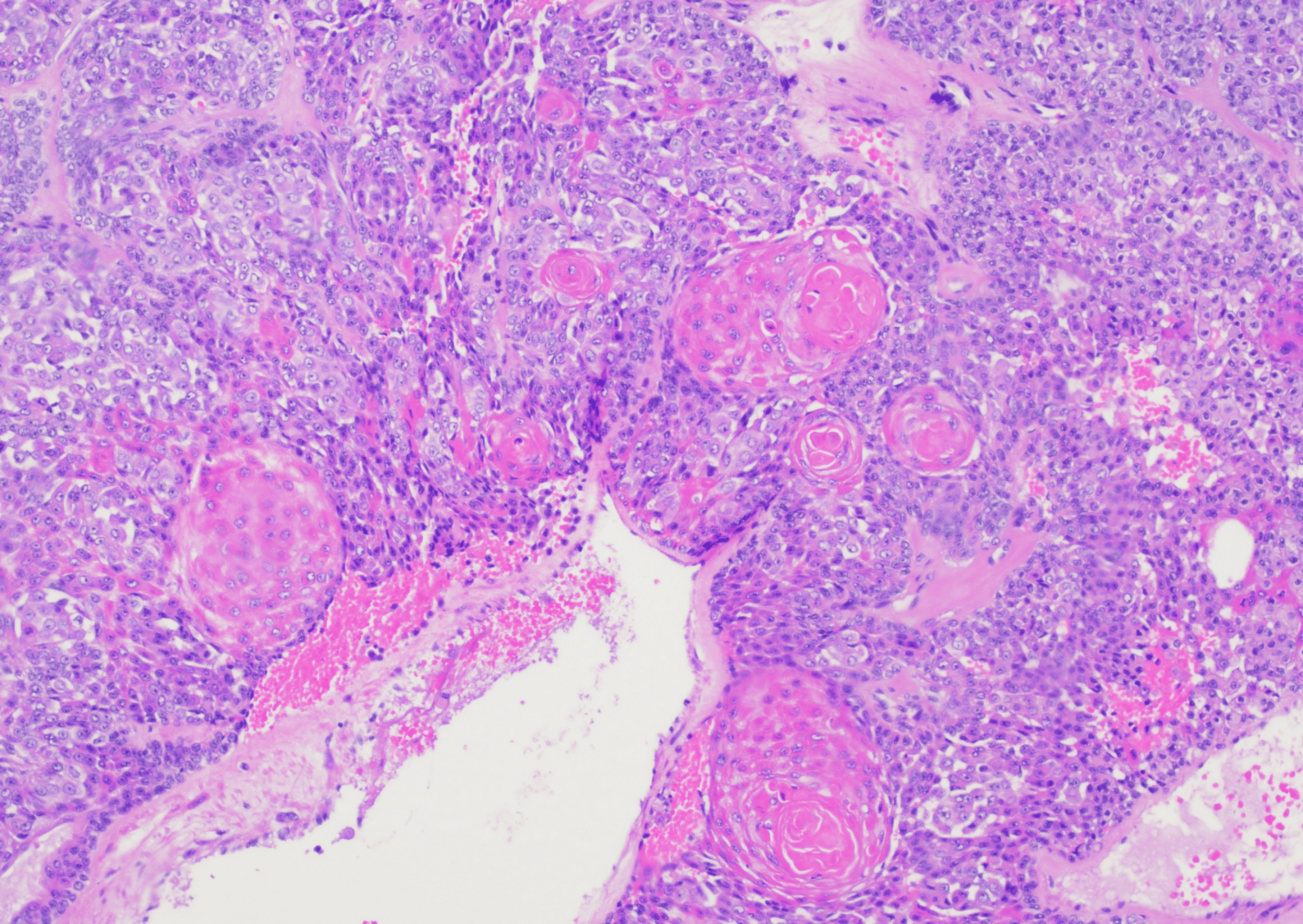
SEDC at low (100x) magnification showing multiple infiltrative nests of atypical squamoid, basaloid, and clear-staining epithelioid cells with focal intervening sclerotic stroma. SEDC, squamoid eccrine ductal carcinoma

**Figure 3:**
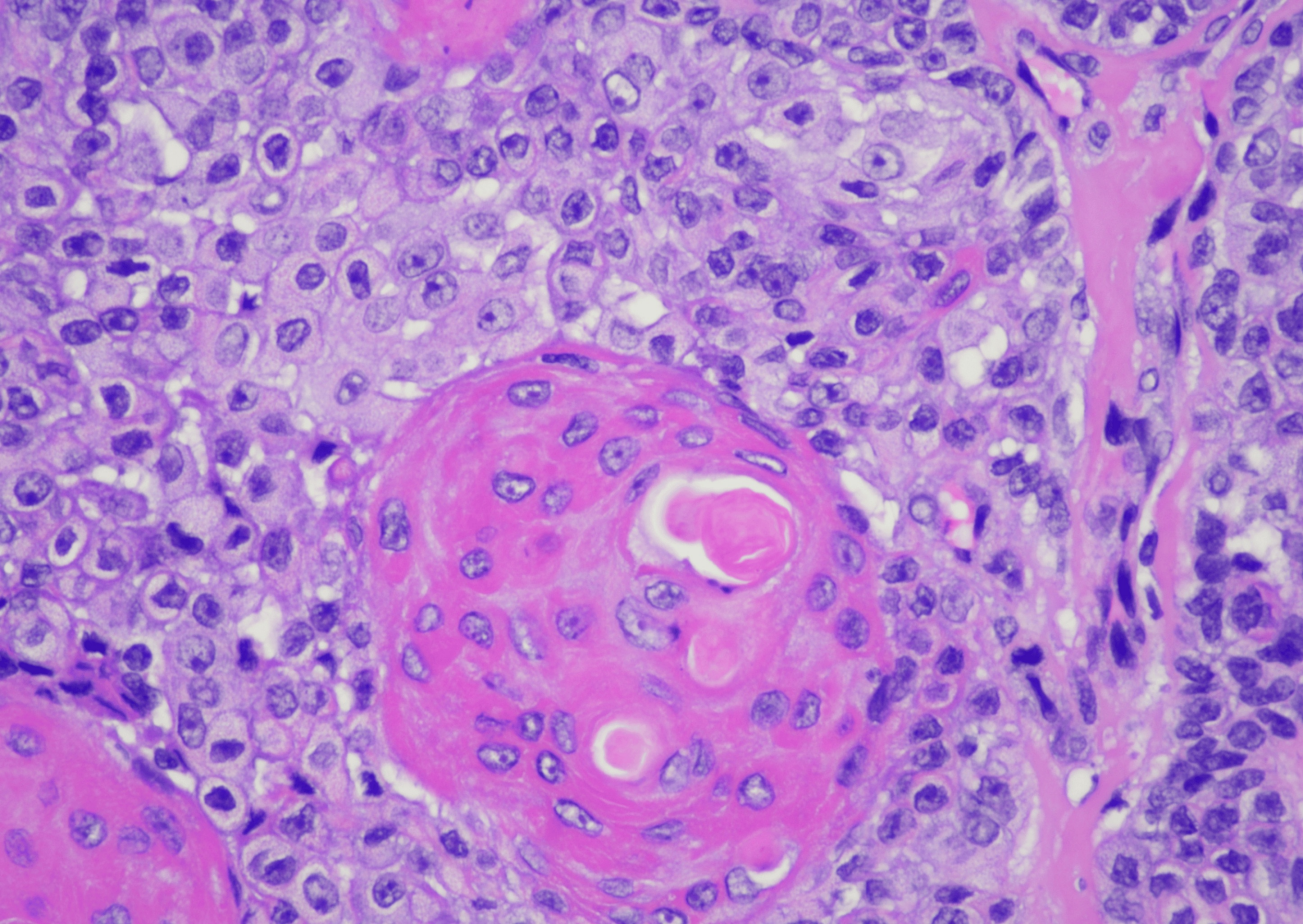
SEDC at high (400x) magnification showing enlarged pale squamoid cells, large cells with abundant eosinophilic cytoplasm, and few smaller basaloid cells surrounding well-formed small ductal lumen and larger keratinizing cystic spaces. The nuclei appear atypical and moderately pleomorphic with irregular nuclear contours and prominent nucleoli. SEDC, squamoid eccrine ductal carcinoma

**Figure 4:**
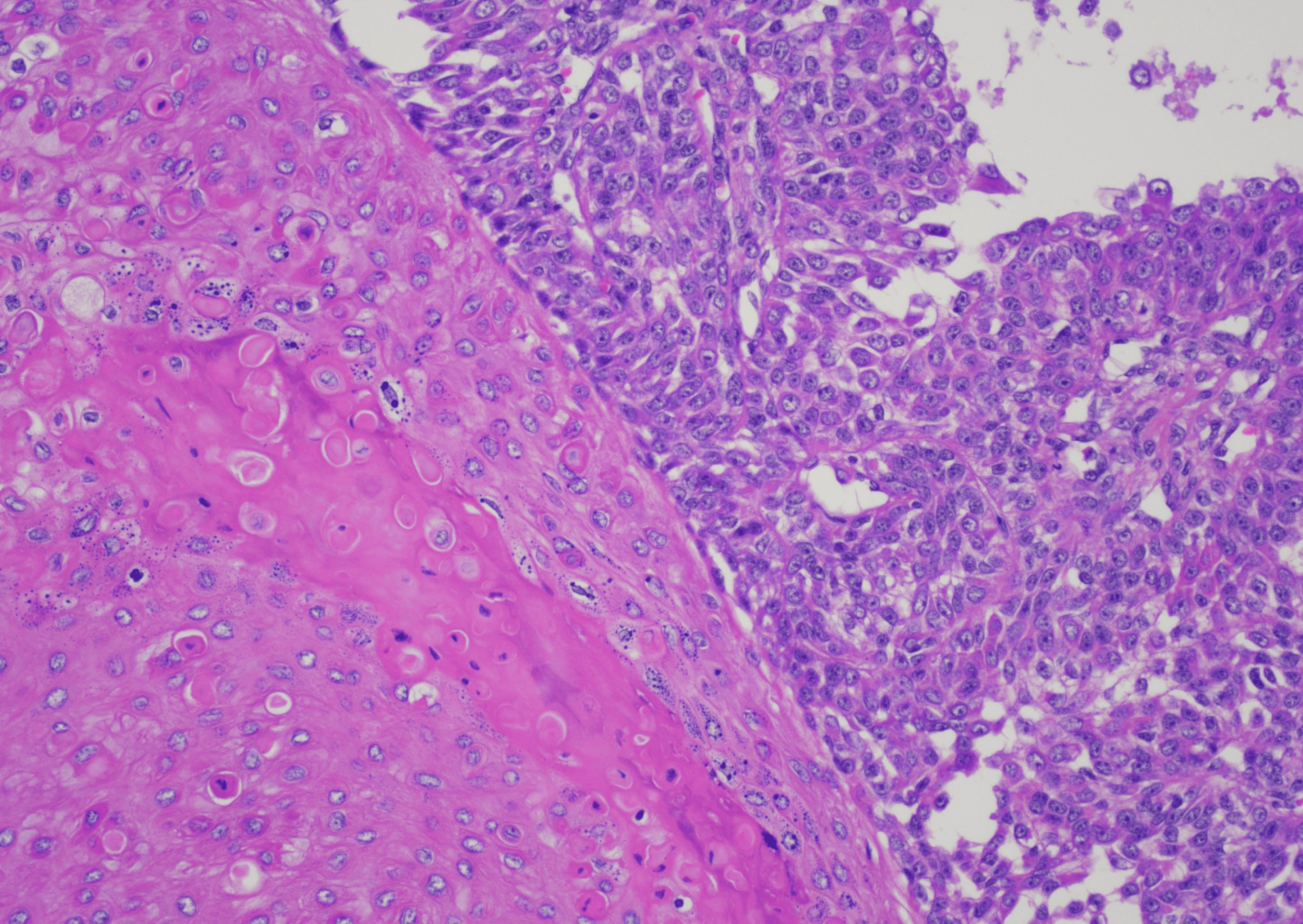
SEDC at moderate (200x) magnification showing prominent interfacing of deeply infiltrating atypical squamoid cells and smaller basaloid cells with numerous small keratinizing ductal lumina and focal dilated cystic spaces. SEDC, squamoid eccrine ductal carcinoma

**Figure 5:**
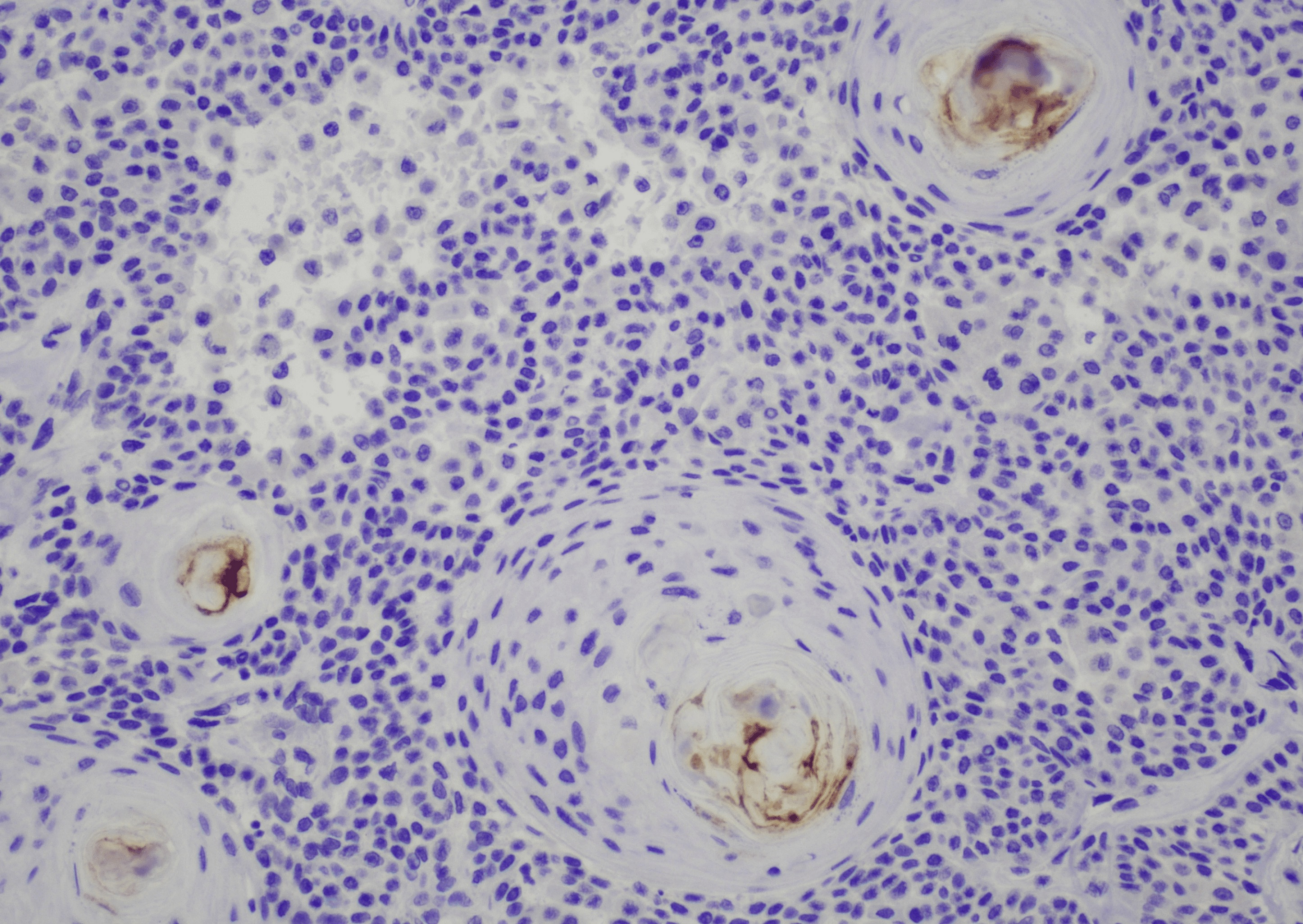
Immunohistochemistry for polyclonal CEA at moderate (200x) magnification shows focal positive staining within ductal lumina. CEA, carcinoembryonic antigen

A 34-year-old Hispanic female gravida 1 para 0 at 37 weeks 0 days gestation by the stated estimated delivery date (EDD) presented to labor and delivery triage for evaluation of a right thigh abscess. The patient had recently transferred prenatal care from an outside clinic two weeks prior to our facility with minimal medical records. Her medical history up until this point was unremarkable. She noticed what she believed was an ingrown hair appearing about four weeks prior. The lesion became larger and more painful after two weeks, and the patient attempted to drain the lesion herself at home, describing the drainage as dark red blood. She reported afterward the area was warm to the touch and irritated. The patient was nontoxic on initial examination and had normal vital signs, so blood work was not collected. The lesion was described as a ~3x3 cm mildly erythematous papule with central fluctuance and peripheral induration. No surrounding erythema was noted, although the tissue subjectively felt warm. Differential diagnoses included abscess, organized hematoma, and cutaneous neoplasm. Given abscess was the initial leading diagnosis, an incision and drainage procedure was performed. Old, thickened blood was expressed from the lesion, along with a membranous material that was sent to pathology. The wound was cleaned with sterile saline and hydrogen peroxide, and the patient was started on a 10-day course of cephalexin 250 mg four times daily (QID). It should be noted that a wound culture was collected at the time as well; however, due to a miscommunication with orders, the culture was never taken to the lab and was accidentally discarded. The patient was evaluated in the gynecology clinic six days later where the lesion had healed over and the appearance remained unchanged (Figure 1). Given that the pathology results were still pending, and wound culture was unavailable, the decision was made to extend the duration of antibiotics for a total of 14 days. After completing the antibiotic course, the lesion did appear improved compared to the initial presentation and was not opened again due to concerns of causing a superimposed infection. Eighteen days after the initial presentation and biopsy, the pathology report returned confirming the diagnosis of SEDC, and dermatology was consulted the same day for further recommendations (see Figures 2, 3, 4, 5 for pathology images and descriptions). Given the rarity of the disease, no standard treatment course has been established. Dermatology reviewed a case series for treatment guidelines and subsequent outcomes, ultimately recommending removal of the lesion with either a wide local excision (WLE) or Mohs surgery. No recommendation currently exists for sentinel lymph node biopsy, and dermatology did not recommend further imaging to rule out metastasis. Expedited delivery was not indicated. 

The patient was promptly seen by dermatology three days after diagnosis and plans were made for a WLE procedure. While at this visit, two more lesions, described as 6 mm firm flesh-colored papules, were also identified on the patient’s right and left thighs and biopsies were taken. The right papule was identified as a dermatofibroma, while the left sided papule was identified as a seborrheic keratosis. Three days after her appointment with dermatology, the patient went into spontaneous labor and underwent a forceps assisted vaginal delivery. Postpartum course was unremarkable. The patient was seen by dermatology 10 days after delivery and underwent an uncomplicated WLE procedure of the lesion, with final pathology consistent with initial biopsy results and negative margins. On a follow-up exam in the dermatology clinic, sutures were removed, and the wound was determined to be healing appropriately. 

## Discussion

SEDC is characterized as an invasive dermal tumor, comprising lobules, nests, and cords of cells with an infiltrative appearance (Figure 2). This tumor consists of a heterogeneous population of cells, including larger squamoid cells and smaller basaloid adnexal cells [7]. The squamoid cells exhibit more prominent nucleoli and copious eosinophilic to amphophilic cytoplasm, and the adnexal cells are identified by their diminutive, hyperchromatic nuclei, and limited cytoplasm (Figure 3) [3]. Both cell types are often observed encasing ductal lumina of variable size. Additionally, signs of follicular differentiation may be observed, which include occasional infundibulocystic keratinizing structures and external root sheath differentiation (Figure 4). This follicular differentiation in SEDC has led to the hypothesis that it might represent a squamoid variant of microcystic adnexal carcinoma (MAC), which can resemble SEDC histologically [8]. MAC lacks squamous eddies and tends to display more frequent ductal structures with atypical cells [9]. Other frequently considered histologic differential diagnoses include squamous cell carcinoma (SCC), eccrine syringoid carcinoma, and eccrine porocarcinoma [10]. SEDC may be distinguished from SCC by the absence of actinic keratosis or SCC in situ as well as the absence of high-grade atypia [11]. Eccrine syringoid carcinoma is characterized by ductal proliferation in the deeper layers of the dermis, but it does not exhibit squamous or follicullocystic differentiation. Porocarcinoma, on the other hand, may display squamoid differentiation, but it characteristically involves the overlying epidermis as well, a feature not commonly seen in SEDC.

The rarity of SEDC, combined with high rates of misdiagnosis, makes identifying this disease quite challenging, even for experts in the field [5]. The appearance of lesions tends to vary widely and have no distinct features, although available reports in the existing literature have described them as ulcerated plaques or nodules [11]. Furthermore, given the rarity of the disease, there is no consensus on treatment guidelines. WLE appears to be the modality of choice among available case reports. In one review of 50 case reports, 43/50 were treated with WLE, with a recurrence rate of 26%. 3/50 were treated with Mohs micrographic surgery (MMS), and three cases with recurrence after WLE were also treated with MMS. No known recurrences were seen after MMS [9]. 

This case was particularly unusual given its presentation in a young pregnant woman, which is far from the more typical presentation that is seen in elderly, more often male, individuals [4]. In addition, current literature reports 27 mm as the largest tumor size, with an average time of a few months to up until 10 years before initial biopsy [3]. Rather, our patient noticed the lesion only four weeks prior to initial presentation, indicating rapid growth, and with a tumor size of 30x30 mm, this would be the largest known SEDC tumor to date. Thankfully, the rapid growth of the lesion allowed for early detection, diagnosis, and treatment. For obstetrics providers, who at many times are functioning as both pregnancy providers and primary care providers for reproductive aged women, this case highlights the importance of keeping broad differential diagnoses when evaluating non-obstetric complaints. The decision to biopsy the lesion proved immensely helpful in arriving at the final diagnosis and demonstrates the utility of a simple biopsy when faced with uncertainty. For dermatologists, this case demonstrates that this diagnosis can be made in young, otherwise healthy women, and that treatment of SEDC should not be modified or delayed in the setting of pregnancy.

## Conclusions

SEDC is a rare dermatologic malignancy with no reported cases in pregnancy to date. Our case highlights the unique presentation of this rapidly growing skin lesion and the importance of the obstetrics provider’s recognition of abnormal, concerning skin lesions leading to early diagnostic workup and subsequent treatment by dermatologists, with no delays in the setting of pregnancy. It also highlights the importance of attention to detail of histological findings to establish an accurate diagnosis. This case report adds to the growing, yet still limited literature on this rare diagnosis.
